# Pharmacogenetics of methylphenidate in childhood attention-deficit/hyperactivity disorder: long-term effects

**DOI:** 10.1038/s41598-017-10912-y

**Published:** 2017-09-04

**Authors:** Clara I. Gomez-Sanchez, Juan J. Carballo, Rosa Riveiro-Alvarez, Victor Soto-Insuga, Maria Rodrigo, Ignacio Mahillo-Fernandez, Francisco Abad-Santos, Rafael Dal-Ré, Carmen Ayuso

**Affiliations:** 1 Department of Genetics, IIS - Fundación Jiménez Díaz University Hospital (IIS-FJD, UAM). Avda. Reyes Católicos, 2, Madrid, 28040 Spain; 2 Centre for Biomedical Research on Rare Diseases (CIBERER). C/ Monforte de Lemos 3-5, Pabellón 11, Madrid, 28029 Spain; 3 Department of Psychiatry, IIS - Fundación Jiménez Díaz University Hospital (IIS-FJD, UAM). Avda. Reyes Católicos, 2, Madrid, 28040 Spain; 4 Department of Pediatrics, IIS - Fundación Jiménez Díaz University Hospital (IIS-FJD, UAM). Avda. Reyes Católicos, 2, Madrid, 28040 Spain; 5 Epidemiology Unit, IIS - Fundación Jiménez Díaz University Hospital (IIS-FJD, UAM). Avda. Reyes Católicos, 2, Madrid, 28040 Spain; 6 Clinical Pharmacology Department, IIS- La Princesa University Hospital (IIS-IP). C/ de Diego Leon, 62, Madrid, 28006 Spain; 7 Clinical Research, BUC (Biosciences UAM + CSIC) Program, International Campus of Excellence, Universidad Autónoma de Madrid. Ciudad Universitaria de Cantoblanco, Madrid, 28049 Spain

## Abstract

Attention-deficit/hyperactivity disorder (ADHD) is a common neurodevelopmental disorder in which a significant proportion of patients do not respond to treatment. The objective of this study was to examine the role of genetic risk variants in the response to treatment with methylphenidate (MPH). The effectiveness of MPH was evaluated based on variations in the CGI-S and CGAS scales over a 12-month treatment period using linear mixed effects models. A total of 208 ADHD patients and 34 polymorphisms were included in the analysis. For both scales, the response was associated with time, extended-release MPH/both formulations, and previous MPH treatment. For the CGI-S scale, response was associated with *SLC6A3* rs2550948, *DRD4* promoter duplication, *SNAP25* rs3746544, and *ADGRL3* rs1868790. Interactions between the response over time and *SLC6A3* and *DRD2* were found in the CGI-S and CGAS scales, respectively. The proportion of the variance explained by the models was 18% for the CGI-S and 22% for the CGAS. In this long-term study, the effects of *SLC6A3, DRD4*, *SNAP25*, and *ADGRL3* on response to treatment reflect those observed in previous studies. In addition, 2 previously unreported interactions with response to treatment over a 12-month period were found (*SLC6A3* and *DRD2*).

## Introduction

Attention-deficit/hyperactivity disorder (ADHD) is a very common neurodevelopment condition in children, involving about 5% of children and adolescents^1^. About 65% of ADHD children are also symptomatic in adulthood, thus suggesting that the disease is chronic^2, 3^. The symptoms of ADHD include inappropriate levels of attention and/or hyperactivity and impulsivity. In addition, over 65% of ADHD patients present psychiatric comorbidities, such as depression, anxiety, and learning disorders^4, 5^, all of which affect academic performance and family life, with huge social and economic repercussions^6, 7^.

Stimulants are the most effective medications for improvement of ADHD symptoms, and methylphenidate (MPH) is often the first choice owing to its effectiveness and safety, as demonstrated in several studies^8–10^. However, although the clinical condition of most patients treated with MPH improves, a considerable proportion (35%) do not respond to treatment or present adverse effects, thereby making response to MPH variable and difficult to predict^11, 12^. As a result, clinicians often use a trial-and-error approach based on different types of medication or on titration of dosages to find the best fit for each patient^13^. It seems clear that identifying accurate predictors of response to medication would be beneficial for clinical practice^14–16^.

ADHD is a heterogeneous and complex disorder involving environmental and genetic risk factors. The strong genetic component of ADHD is supported by family, twin, and adoption studies, which have found a mean estimated heritability of 76%^17^, suggesting that ADHD is among the most heritable neuropsychiatric disorders.

Predictions based on genetic factors are the basis of pharmacogenetic testing. Numerous candidate genes have been associated with an increased risk of ADHD^18^. As many of these genes play a role in the mechanisms of action of psychostimulants, there is a high probability that they are also associated with response to treatment^19^. The results of pharmacogenetic studies of ADHD are variable and inconclusive^19^. The objective of this study was to examine the role of risk genes in the response to MPH in children with ADHD and to evaluate the effectiveness of the drug over 12 months of follow-up.

## Results

A total of 238 Caucasian ADHD patients were included in the initial step of the study. After the quality control procedure, 208 patients remained in the final analysis, and 176 completed the 12-month follow-up. Fifty-seven (27%) were treatment-naive. Among the MPH-experienced patients (151), 41% reported a poor or partial response at the time they entered in the study. The demographic and clinical characteristics of the cohort are shown in Table 1.

**Table 1:** Demographic and clinical characteristics of ADHD patients.

	Baseline	3 months	6 months	12 months
**N**	208	172	183	176
**Age**				
Mean (SD)	10.6 (2.9)	10.7 (2.9)	10.5 (2.8)	10.5 (2.8)
Range	6–18	6–18	6–18	6–18
**Gender**				
Male (%)	163 (78.3)	136 (79.1)	145 (79.2)	138 (78.4)
Female (%)	45 (21.7)	36 (20.9)	38 (20.8)	38 (21.6)
**ADHD diagnosis**				
Combined type (%)	121 (58.2)	99 (57.7)	106 (57.9)	105 (59.6)
Inattentive type (%)	78 (37.5)	66 (38.4)	69 (37.7)	65 (36.9)
Hyperactive type (%)	9 (4.3)	7 (4.1)	8 (4.4)	6 (3.5)
**Previous treatment**				
Naive patients (%)	57 (27.4)	47 (27.3)	46 (25.1)	47 (26.7)
Experienced patients (%)	151 (72.6)	125 (72.7)	137 (74.9)	129 (73.3)
**Methylphenidate**				
Immediate-release (%)	17 (8.2)	13 (7.6)	8 (4.4)	8 (4.5)
Extended-release (%)	173 (83.1)	144 (83.7)	155 (84.7)	143 (81.3)
Both at the same time (%)	18 (8.7)	15 (8.7)	20 (10.9)	25 (14.2)
**Doses mg/day**				
Mean (SD)	35.5 (15)	39.5 (15.7)	39.4 (15.8)	40.5 (15.5
**Cases with at least one side effect (%)**		51 (29.6)	72 (39.3)	47 (26.7)
**CGI-S score**				
Mean (SD)	3.24 (0.58)	3.10 (0.56)	3.06 (0.55)	3.07 (0.57)
**CGAS score**				
Mean (SD)	69.15 (11.67)	74.62 (9.26)	75.26 (9.69)	75.90 (9.29)

For all variants, the call rates of genotype per-marker were higher than 96%; therefore, no polymorphisms were excluded from the analysis. The fixed effects of the models are summarized in Table 2 with the point estimate, 95% confidence intervals (95% CI), and p values.

**Table 2:** Significant results of fixed effects from linear mixed-effect models to evaluate the association between covariates/genetic variants and response according to the CGI-S and CGAS scales.

Variable	Genotype	β (95% CI)	Model	P
**CGI-S**				
Time	—	−0.01 (−0.02 to −0.01)	—	<0.001
Previously treated patients	—	−0.25 (−0.40 to −0.12)	—	0.001
Extended-release MPH or both formulations	—	−0.38 (−0.58 to −0.20)	—	<0.001
*SLC6A3* rs2550948	A/G o G/G	−0.14 (−0.27 to 0.02)	Dominant	0.011
*DRD4* promoter duplication	S/S	0.74 (0.40–1.18)	Recessive	0.001
*SNAP25* rs3746544	A/C o C/C	−0.16 (−0.27 to −0.03)	Dominant	0.018
*ADGRL3* rs1868790	A/A	0.23 (0.02–0.45)	Recessive	0.026
Interaction				
*SLC6A3* intron 8 VNTR *Time	6/− o −/−	−0.02 (−0.03 to −0.01)	Dominant	0.010
**CGAS**				
Time	—	0.51 (0.39–0.60)	—	<0.001
Treatment-experienced patients	—	5.15 (2.57–7.73)	—	<0.001
Extended-release MPH or both formulation	—	7.74 (4.54–10.94)	—	<0.001
Dosage	—	0.03 (0.02–0.09)	—	0.008
Interaction				
*DRD2* rs1800497*Time	T/T	−0.63 (−1.17 to −0.08)	Recessive	0.024

Sex, age, and ADHD subtype were not significant as covariates for any of the efficacy models. We found a significant improvement in the response over time in the CGI-S and CGAS scales. In addition, previous MPH treatment and extended-release MPH alone/both formulations were positive predictors of response according to CGI-S and CGAS. Dosage was a significant factor only in the CGAS effectiveness model.

**Figure 1:**
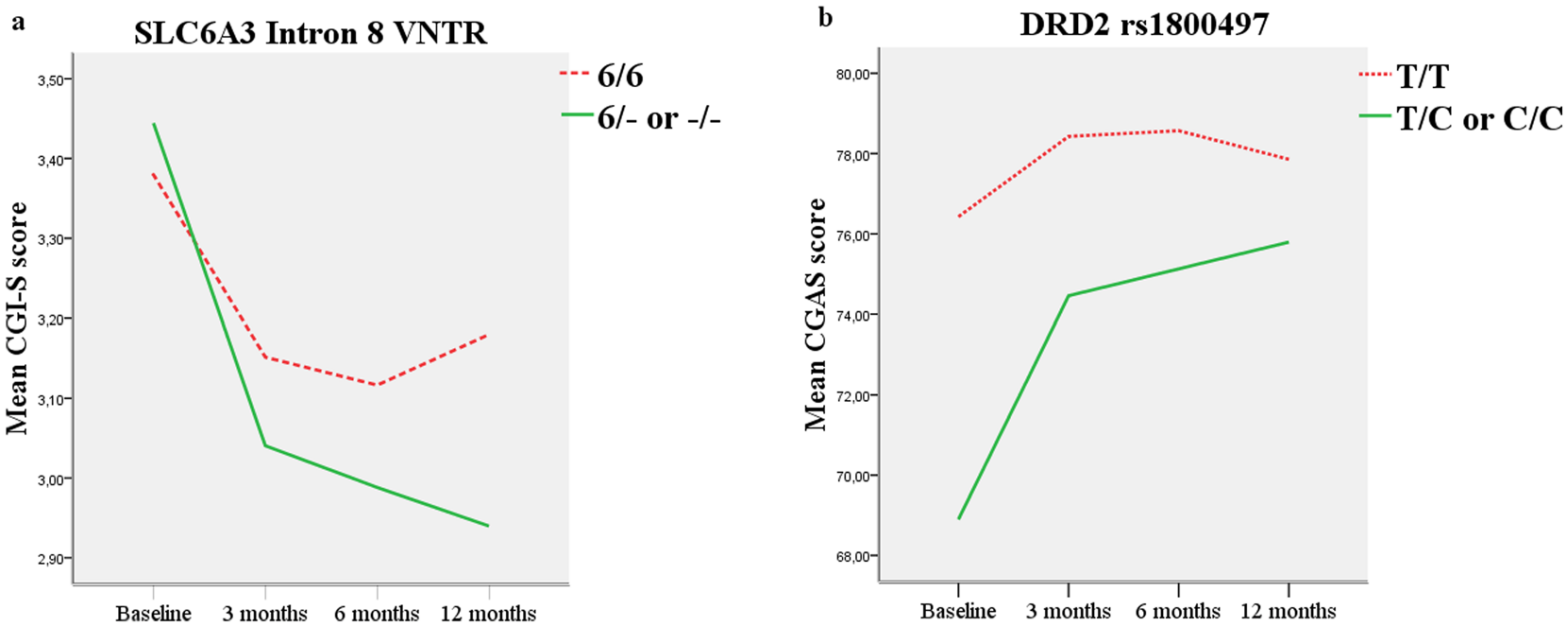
Significant interactions between response over time and genetic variants in the CGI-S model (**a**) and CGAS model (**b**).

As for the genetic component, in the CGI-S model, we found recessive effects in *DRD4* promoter duplication, and *ADGRL3* rs1868790, which were associated with significant impairment, and a dominant effect in *SLC6A3* rs2550948 and *SNAP25* rs3746544, with a significant improvement in the symptoms. Moreover, an interaction was found between the *SLC6A3* intron 8 VNTR and treatment over time (Table 2 and Fig. 1).

Finally, no significant association was observed between CGAS scores and genetic variants, although an interaction was found between *DRD2* rs1800497 and treatment over time (Table 2 and Fig. 1).

The proportion of the variance explained by the model was 18% for CGI-S and 22% for CGAS. The datasets generated during and/or analysed during the current study are available from the corresponding author on reasonable request.

## Discussion

We investigated the role of genetic factors that were associated with ADHD as predictors of the clinical response to MPH in a Caucasian population; the interaction between genetic variants and treatment over time was also assessed.

The values for CGI-S and CGAS improved significantly over time, although with a modest effect size, maybe because 73% of patients were on treatment before the beginning of the study. ADHD subtype was not a significant factor in response to treatment, according to previously studies^20^. In addition, treatment-experienced patients and patients who took extended-release MPH or both formulations had a better response in all the efficacy parameters considered (CGI-S and CGAS scales). Treatment-experienced patients were better responders, possibly because of selection bias: they continued on treatment because they were responding to the treatment, with no safety issues. Adjusting models by treatment status, we controlled which polymorphisms were associated with the response regardless of the confounding variable. The resulting data suggest that the extended-release formulation could have led to an improvement in the response to MPH, for example, by improving adherence to treatment^21^. Dosage was only a significant factor in the CGAS model. In CGI-S, statistical significance may not have been reached because of insufficient sample size.

As for the genetic component, our results showed the implication of the genes *SLC6A3* and *DRD4*, which have been widely associated with changes in response to MPH^19, 22^. On the other hand, we failed to replicate the most studied variant in *SLC6A3* (VNTR 3′ UTR), thus reflecting previous negative results on the implication of this variant and the pharmacogenetics of ADHD^19, 23^. Consequently, other polymorphisms of this gene must be explored. In this context, we found a previously unreported association between a promoter variant (rs2550948) and response to MPH. In the case of *DRD4*, once again, we did not replicate the most studied association, which was with the VNTR exon 3 variant^19^. The results of studies that investigate the implication of this variant in response to MPH are also conflicting^19^. However, we did find an association between the *DRD4* tandem duplication polymorphism in the promoter region and response to MPH. No significant effect on response to MPH was found with this variant in 2 small-scale studies of children^24^ and adults^25^.

A statistically significant association with response to MPH was found in the neurodevelopmental genes *SNAP25* and *ADGRL3*. Although the role of these genes in susceptibility to ADHD is widely studied, they have been less largely studied in terms of the effectiveness of MPH. Contini *et al*.^26^ evaluated the same polymorphism in *SNAP25* (rs3746544) but identified no effect in adults with ADHD. Elsewhere in the literature, inconsistent findings were found between *ADGRL3* and response to MPH. The marker rs6551665 had previously been associated with the response to MPH^27, 28^. Arcos-Burgos *et al*.^27^ reported that the G allele was associated with a good response, whereas Labbe *et al*.^25^ found that it was associated with a poor response. The divergent results could be explained by differences in sample subtypes or outcome measures^29^. In addition, the marker rs6858066 was associated with a better response^28, 30^. In the present study, the associations between rs6551665 and rs6858066 and response to MPH were not statistically significant. In contrast, we established a previously unreported statistically significant association between rs1868790 and response to MPH.

Furthermore, our pharmacogenetic study suggested that *SLC6A3* and *DRD2* genotypes were associated with different degrees of improvement in ADHD symptoms. For both genes, a faster response effect was observed during the first 3 months, respectively, in patients with the genotypes 6/− or −/− and T/C or C/C. To our knowledge, this is the first report of differences in the response to MPH over time in these genes.

Our study reflects the considerable difficulty in replicating pharmacogenetic association studies in ADHD. The results reported are conditioned by polymorphisms analyzed per gene or model of inheritance evaluated. Furthermore, they depend on factors that influence the response, such as phenotype, concomitant treatment, and sample characteristics (age, sex, ADHD subtype), which are not always taken into account^19, 31, 32^. Results are also difficult to reproduce because of the definition of response in assessment scales. In fact, there is no clear consensus on the best approach to find objective and reliable measures of response to treatment^33^.

The strengths of our study are that we evaluated response using 2 scales that provide more detailed information and thus reveal the heterogeneity in response effect. Moreover, we evaluated the response to MPH under conditions of routine clinical practice, thus highlighting the role of genetic factors in real-world situations^34^. Ours is the first 12-month study of the pharmacogenetic of response to MPH in children, and we provide much more relevant clinical information than short-term studies. The literature contains little evidence of the long-term effects of medication owing to the difficulty in follow-up and the low persistence on therapy rate^35^.

Some limitations of this study should be considered. First, the determination of the scores for the clinical response through the scales CGI-S and CGAS, which are recorded by doctors, despite are based on what parents and children report, is not free of subjectivity risk of the doctor. However, having scales assessing what parents and patients believe with regards to the overall functioning of patients would be of interest in the assessment of the treatment effectiveness, since it would be free of evaluator’s bias^36^. By contrast, this approach has to cope with the risk of serious discrepancies between informants, which ultimately will hinder patient’s assessment^37^.

Another limitation of the study to be considered is that 73% of patients were on treatment before beginning the study. Although we have adjusted the models by previous treatment status, experienced patients started with better response at baseline visit, and for that reason they had limited clinical improvement at the end of the study period.

In conclusion, we report moderate effects of the genes *SLC6A3, DRD4*, *SNAP25*, and *ADGRL3* in the response to MPH, thereby supporting several previous studies of these genes. We also found interactions between response to treatment over 12 months and genotypes of *SLC6A3* and *DRD2*. When all the covariates are taken into account, the models explain around 20% of the response to MPH. Therefore, other genetic or non-genetic factors must be involved in the variability of response to MPH. More research is required to find pharmacogenetic variants that could help to establish the best treatment regimen.

## Method

### Patients, clinical assessment, and ethical review

We performed a prospective, observational study of unrelated Spanish Caucasian patients with ADHD aged 6 to 18 years who were enrolled and clinically assessed by psychiatrists and pediatricians at Fundación Jiménez Díaz University Hospital. ADHD was diagnosed following the Diagnostic and Statistical Manual of Mental Disorders^38^. All patients were evaluated, taking into account different sources of information (parents, children and clinicians).

To be included, patients had to have ADHD, be Spanish and Caucasian, be treatment-naive or have been treated with only MPH at baseline, and have been receiving MPH at least from baseline onwards. Patients could receive one of 2 formulations: (a) the immediate-release formulation (Rubifen) or (b) the extended-release formulation (Medikinet and Concerta)/ both formulations. Patients treated with medication (in addition or instead of) other than MPH were excluded.

Doses and type of MPH formulation were individually prescribed according to the summary of product characteristics and the clinical criteria of the psychiatrist and were adjusted during follow-up visits until the desired therapeutic effects were obtained.

Clinical effectiveness was evaluated using the Clinical Global Impression-Severity (CGI-S) scale^39^ and the Children’s Global Assessment Scale (CGAS)^40^. CGI-S provides a global evaluation of the severity of illness at the time of evaluation using a 7-point scale ranging from 1 (no impairment, normal) to 7 (maximum impairment). CGAS is used to rate the general functional status in children and adolescents using a numerical scale, with values ranging from 1 (need for constant supervision) to 100 (superior functioning).

During the assessment period, the following side effects were evaluated: loss of appetite, insomnia, gastrointestinal problems, headaches, cognitive, emotional and behavioral disturbances.

Clinical assessments were performed at baseline and after 3, 6, and 12 months of treatment with MPH.

The study protocol was reviewed and authorized by the Research Ethics Committee of the IIS-Fundación Jiménez Díaz University Hospital. The study was carried out in accordance with the ethical principles that are reflected in the Declaration of Helsinki. Before recruitment, once the study objectives and procedures had been detailed, parents or legal guardians signed a written informed consent.

### DNA extraction and genotyping

Peripheral blood lymphocytes or saliva were used to obtain genomic DNA, employing an automatic DNA extractor (BioRobot EZ1, Qiagen, Hilden, Germany) or the Oragene DNA self-collection kit (DNA Genotek, Kanata, Ontario, Canada), respectively, according to the manufacturer’s recommendations. DNA concentration and sample quality were evaluated through a spectrophotometer (NanoDrop® ND-1000 Spectrophotometer, Wilmington, DE, USA).

Thirty-four polymorphisms from 18 genes were chosen according to their significance in the literature. All genes were previously associated with ADHD (Table 3).

**Table 3:** Description of the genes and polymorphisms analyzed.

Gene	Description	Variant	Reference
*SLC6A2*	Norepinephrine transporter	rs28386840^a^	44
		r5569^c^	18
*ADRA2A*	Adrenergic receptor alpha 2A	rs1800544^a^	18
		rs553668^e^	18
*SLC6A3*	Dopamine transporter	rs2550948^b^	45
		rs2652511^b^	45
		rs11564750^a^	45
		3’UTR VNTR^e^	18
		Intron8 VNTR^d^	18
*DRD2*	Dopamine receptor D2	rs1800497^f^	46
*DRD4*	Dopamine receptor D4	rs3758653^a^	47
		Exon3 VNTR^c^	46
		Promoter duplication^b^	46
*SLC6A4*	Serotonin transporter	Promoter VNTR^b^	18
		Intron2 VNTR^d^	18
*HTR2A*	Serotonin-2A receptor	rs7322347^d^	48
*HTR2C*	Serotonin-2C receptor	rs6318^c^	49
*GRM7*	Glutamate receptor metabotropic 7	rs3792452^d^	50
*SLC9A9*	Glycine transporter	rs9810857^f^	51
*COMT*	Catechol-O-methyltransferase	rs4680^c^	18
		rs4818^c^	52
*SNAP25*	Synaptosomal-associated protein 25kDA	rs3746544^e^	18
*DDC*	Dopa decarboxylase	rs6592961^d^	48
*STS*	Steroid sulfatase	rs12861247^d^	53
		rs17268988^d^	53
*FADS2*	Fatty acid desaturase 2	rs498793^d^	54
*ADGRL3*	Adhesion G protein-coupled receptor L3	rs1397548^c^	27
		rs2305339^d^	27
		rs6551655^d^	27
		rs1868790^d^	30
		rs6813183^d^	30
		rs6858066^d^	30
*CDH13*	Cadherin 13	rs6565113^d^	47
*GFOD1*	Glucose-fructose oxidoreductase domain containing 1	rs552655^d^	47

Position in the gene: ^a^Upstream variant, ^b^Promoter variant, ^c^Exon variant, ^d^Intron variant, ^e^3′UTR variant, ^f^Downstream variant.

Single-nucleotide polymorphisms (SNPs) were genotyped through the TaqMan on-demand or pre-designed SNP genotyping assays system, according to the company’s instructions (Applied Biosystems, Foster City, CA, USA). We run PCR and allelic discrimination assays in a LightCycler 480 (Roche Diagnostics, Mannheim, Germany) and we analyzed them using the LightCycler^®^ 480 software, version 1.5. (Roche Diagnostics, Mannheim, Germany).

Variable number tandem repeat (VNTR) polymorphisms were identified using fragment analysis. PCR products were displayed on an ABI Prism 3130xl DNA sequencer (Applied Biosystems Foster City, CA, USA), and we analyzed the results by means of GeneMapper software, version 4.0 (Applied Biosystems, Foster City, CA, USA). Primer sequences and PCR conditions can be provided upon request.

For each VNTR polymorphism, subjects were categorized into 3 genotypes according to the previously described risk allele^18^, as follows: *SLC6A3* 3′UTR VNTR (10/10, 10/−, −/−), *SLC6A3* intron8 VNTR (6/6, 6/−, −/−), *DRD4* promoter duplication (L/L, L/S, S/S), *DRD4* exon3 VNTR (7/7, 7/−, −/−), *SLC6A4* promoter VNTR (L/L, L/S, S/S), and *SLC6A4* intron2 VNTR (10/10, 10/−, −/−).

### Statistical analysis

During the quality control procedure, genotype call rates per sample and per polymorphism < 80% were excluded from the analysis. The outcome measures of treatment with MPH were evaluated as quantitative data according to CGI-S and CGAS. Analyses of the effects of different genotypes on response to treatment over time were performed using linear mixed-effects models. These mixed-models are useful for repeated-measures analyses where follow-up times are not uniform across all subjects^41^. Models were constructed using the lme function from the nlme package in R.

As in other genetically complex diseases in which the model of inheritance is uncertain, the analyses were performed under the assumption of dominant, recessive, codominant, and additive models. The best model was selected based on the one with the lowest Akaike information criterion (AIC). Data for variants located on chromosome X (*HTR2C* and *STS* genes) were analyzed taking X inactivation into account according to Clayton’s approach^42^.

Age, sex, ADHD subtype, previous treatment, type of MPH (immediate-release vs. extended-release/both formulations), and dosage were also entered into the models as potential explanatory covariates. Statistical significance for main effects and interactions was assessed using the ANOVA F-test and set at a 2-tailed p value of 0.05. In these multivariable models, the effect size of the associations was measured by the coefficients of the models “β”.

The proportion of the variance explained by the model was assessed using Omega Squared^43^.
